# A network-based microfoundation of Granovetter’s threshold model for social tipping

**DOI:** 10.1038/s41598-020-67102-6

**Published:** 2020-07-08

**Authors:** Marc Wiedermann, E. Keith Smith, Jobst Heitzig, Jonathan F. Donges

**Affiliations:** 1 FutureLab on Game Theory & Networks of Interacting Agents, Complexity Science, Potsdam Institute for Climate Impact Research, Member of the Leibniz Association, P.O. Box 60 12 03, 14412 Potsdam, Germany; 2 GESIS — Leibniz Institute for the Social Sciences, Member of the Leibniz Association, Unter Sachsenhausen 6-8, 50667 Cologne, Germany; 3 FutureLab Earth Resilience in the Anthropocene, Earth System Analysis, Potsdam Institute for Climate Impact Research, Member of the Leibniz Association, P.O. Box 60 12 03, 14412 Potsdam, Germany; 4 Stockholm Resilience Centre, Stockholm University, Kräftriket 2B, 114 19 Stockholm, Sweden; 5 Institute of Science, Technology and Policy, ETH Zurich, Zurich, Switzerland

**Keywords:** Complex networks, Computational science

## Abstract

Social tipping, where minorities trigger larger populations to engage in collective action, has been suggested as one key aspect in addressing contemporary global challenges. Here, we refine Granovetter’s widely acknowledged theoretical threshold model of collective behavior as a numerical modelling tool for understanding social tipping processes and resolve issues that so far have hindered such applications. Based on real-world observations and social movement theory, we group the population into certain or potential actors, such that – in contrast to its original formulation – the model predicts non-trivial final shares of acting individuals. Then, we use a network cascade model to explain and analytically derive that previously hypothesized broad threshold distributions emerge if individuals become active via social interaction. Thus, through intuitive parameters and low dimensionality our refined model is adaptable to explain the likelihood of engaging in collective behavior where social-tipping-like processes emerge as saddle-node bifurcations and hysteresis.

## Introduction

Studies of collective behavior or action, such as protest demonstrations, responses to disasters or even revolution^1^, fosters an understanding of the formation and logic of the *crowd*^2–5^. Broadly, the study of collective behavior can be separated into either that of *social movements* or that of *temporary gatherings*. Social movements are usually more structured around specific, identified goals, have deeper social connections between actors, are organized (generally to defend or fight against existing authorities) and persist over time (such as the civil rights movements)^6^. In contrast, gatherings (such as riots, sudden protests, concerts, sporting events) are more spontaneous, less organized, do not carry as deep of social connections between actors, and can be quite ephemeral^7,8^.

Further, individual engagement in collective behaviors (such as changing consumption behavior or adoption of new technologies) can be connected to broader social processes, such as norms and expectations for behavior^9^. Specifically, individuals strategically control their actions in accordance with their norms in order to achieve their goals and objectives^4,5,10^. As such, norms and preferences structure an actor’s likelihood to engage in collective behaviors, as well as its form of participation within these groups. Complex forms of collective behaviors (be it either a movement or a crowd) are thus created through dynamic interactions of actors that share common goals and objectives for a given social situation. For example, global climate change has been frequently noted as one prominent contemporary social problem that could trigger and might also be addressed through collective behaviour (such as the emergent ‘Fridays for Future’^11^ movement)^12–14^.

Empirical evidence for such complex contagion of interlinked individuals leading to collective action has been found for both online^15–17^ and offline^18^ social networks. Additionally, complex contagion has been experimentally shown to foster social tipping^19^, a process that has gained increased attention in the recently^20^ due to its potential for rapid societal changes with profound impacts on the entire socio-ecological Earth System^13,21^. Complementing empirical studies, recent conceptual models of complex contagion incorporate the spreading of an action, behaviour or trait through a complex network^22–26^. They often aggregate an individual’s surrounding over time^27,28^ or abstract space^29^ to accumulate exposure to a considered trait such that at a certain point the individual adopts that trait as well. Such models have been applied successfully to study processes involved in the spreading of opinions^30,31^, large-scale epidemics^24^, the adoption of life-style choices^32^ or the collective behaviour of animal groups^33,34^. However, most such models of collective behavior are often tailored to a specific problem (both in the incorporated processes as well as the underlying parameter set) and are thus often not transferable to different and novel applications.

The *Granovetter threshold model* is a comparatively early contribution to this field, providing a core basis for subsequent and more contemporary modeling attempts^35^. This model aims to explain the emergence of collective behaviors while noting that individual norms and preferences are a crucial factor determining their development and final outcome. In particular, when presented with a simple binary choice – to participate within a collective behavior or not – each individual has a certain activation threshold for participation. This measures the proportion of the group that an individual would like to observe participating within the collective behavior before they are willing to join themselves. The thresholds emerge from the norms, preferences, goals and beliefs of each individual, e.g., representing a kind of trade-off between the costs and benefits of joining in the behavior. As such, the application of the threshold model, or variations thereof, is not limited to simple crowd-like behaviors, such as protests and riots, but is comparatively broad, encompassing collective behaviors e.g., voting^36^, diffusion of innovations^37^, or migration^38^, as well as classical social movements such as the Monday Demonstrations in East Germany^39^. However, while by design the model is very flexible, it has mainly been used for illustrative and theoretical purposes (including most applications outlined above), but hardly applied as a numerical modeling tool.

This paper identifies two major sets of issues that prevent broader application of the Granovetter model and proposes extensions to resolve them. First, under often assumed threshold distributions (such as cut-off Gaussians^35^) the model usually unrealistically predicts either no-one or the entire population to eventually act. We resolve this issue by drawing from real-world observations, social movement and resource mobilization theories^40,41^, as well as recent theoretical and numerical results regarding network spreading processes^42,43^ to extend the original model by classifying individuals as either certainly active, certainly inactive, or contingently active. This causes the model to display nontrivial equilibria in which a certain part of the contingent individuals becomes active. Second, the emergence and shape of the threshold distribution itself is often underexplained. Therefore, we utilize an established conceptual network cascade model^29^ and show that a broad (non-Gaussian) threshold distribution emerges from microscopic networked interactions in which potentially active individuals join an action if a sufficient number of their neighbors are also engaged. We thus specifically acknowledge empirically observed tendencies of individuals to make decisions with respect to their immediate social surrounding rather than considering the entire global population, i.e., the mean field^19,44,45^. By addressing both of the above issues, we effectively separate (unique) individual preferences which determine general tendencies towards or against an action from the embedding of each individual into a larger social structure and corresponding exposure to external influences. Both characteristics then co-determine whether the individual ultimately joins into an action or not.

The remainder of this work is organized as follows. We first introduce the formal specifics of the Granovetter threshold model and discusses in detail its aforementioned conceptual limitations. We then implement the proposed solutions and present a refined threshold model that only depends on parameters that are readily observable in real-world systems. Additionally, we provide an analytical solution of the refined model and analyse its potential for modeling social tipping. Ultimately, we culminate with a discussion of the results and an outlook to future work.

## Granovetter’s threshold model

The threshold model assigns each individual in a population of size *N* a threshold that defines the number of others that must participate in an action before the considered individual does so, too^35^. In its discrete-time formulation the number of acting individuals at time $t+1$, $R(t+1)$, is hence directly derived from the cumulative distribution function of thresholds in the population, *F*, such that

1 $$R(t+1)=NF(R(t)).$$

**Figure 1:**
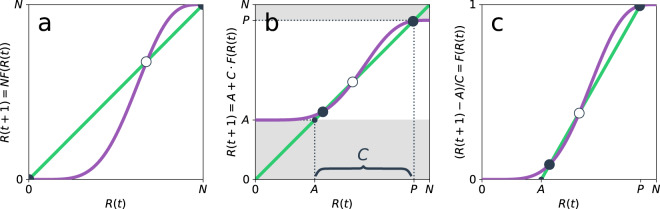
Extension of Granovetter’s (graphic) model with *P* potentially and *A* certainly acting individuals. (**a**) The original model that computes the number of acting individuals $R(t+1)$ from the cumulative distribution function of thresholds $F$. The purple line indicates a typical normal-like choice for this distribution. The 45°-line (green) intersects $F$ at the stable (black) and unstable (white) equilibrium points R*. As for many realistic choices of $F$, only R* = 0 and R* = *N* are stable. (**b**) Introducing *A* certainly and *P* potentially acting individuals, such that the $C=P-A$ contingent individuals have the same threshold distribution $F$ as the entire population $N$. Here, the equilibria move to the interval ${R}^{\ast }\in [A,P]$ and are not necessarily located at exactly ${R}^{\ast }=A$ and ${R}^{\ast }=P$. Hence, the $A$ certainly acting individuals trigger some contingent individuals to act, too. (**c**) Rescaling $R(t+1)$ to the unit interval shows that equilibria can be computed by shifting the diagonal line from crossing $(0,0)$ and $(N,N)$ (as in (**a**)) to crossing $(A,0)$ and $(P,1)$ and using the same threshold distribution *F* as in (**a**).

Note that the original exemplary application of the model was that of individuals’ participation in riots. Hence the choice of the symbol *R* for the number of acting individuals. An equilibrium number of acting individuals R* is obtained by solving $R(t+1)=R(t)=NF(R(t))$ for $R(t)$ which is equivalent to finding an intersection of the graph of *F* with the diagonal through $(0,0)$ and $(N,N)$, Fig. 1a. All equilibrium points R* at which *F* intersects the diagonal line from above are stable, while all others are unstable^35^.

While the threshold model has been widely used within a broad literature^41,46,47^ it has up to now been mainly used for illustrative purposes as a number of issues hinder its application as numerical modeling tool:

### Plausible distributions typically predict no one or the entire population to act

As thresholds are hard to estimate, one typically assumes Gaussian threshold distributions^35^ cut off at the extreme values 0 and *N*. However, assuming a mean threshold *μ* of reasonable size and a moderate standard deviation *σ* implies that there are only few individuals with low or high thresholds and many with medium thresholds close to *μ*. Hence, under the typical assumption of a low number of *instigators*^35^ the model usually predicts zero eventually acting individuals, Fig. 1a. Only if a sufficiently large *σ* is chosen more individuals than the instigators become active. However, the choice of a large *σ* causes the distribution to become rather flat instead of bell-shaped. For example, for a population size of $N=100$ and an average threshold of $\mu =25$, a standard deviation of $\sigma =12.2$ is required so that a single instigator can cause the rest of the population to become active^35^.

In addition, if no individual has a threshold larger than 100%, the threshold model generally has a second typically stable fixed point at ${R}^{\ast }=N$ implying that the entire population has the potential to become active if only enough others do so, too, Fig. 1a. In reality, an individual may never engage in an action regardless of how many others have already joined as personal preferences, norms or attitudes can restrict behaviours^9^. In its basic setup, the Granovetter model can only account for this by either assigning the concerned individuals a threshold of 100% or by selecting the population such that only those individuals that are generally in favour of a certain action are considered^35^. The first approach, however, implies that everyone would generally be willing to act if only enough other individuals become active before. The second approach requires updating the population and, hence, its size, whenever the norms and attitudes of an individual change. What both approaches have in common is that they imply a constant change of the threshold distribution whenever individuals alter their preferences or attitudes.

We therefore propose a framework that refines the threshold model and accounts for the above issues by grouping individuals according to basic preferences that determine whether they certainly, contingently or never act. This circumvents the existence of trivial solutions and we show below that this approach does not require a constant updating of the threshold distribution as a response to changing group memberships.

### The threshold distribution can not be observed, but emerges from microscopic factors

Broadly, two complementary aspects shape whether an individual engages in an action or not. On the one hand there are *individual* factors (such as background characteristics, social class, education or occupation^48,49^), that determine the acceptance of or inclination towards an action. On the other hand there are *group* factors, i.e., characteristics resulting from one’s embedding in a larger social network (such as social position, influence, or peer pressure^50^). Both traits and processes ultimately co-determine the macroscopic threshold that is exposed to the observer and we call these thresholds of the original Granovetter model *emergent thresholds* from here on. However, quantifying the emergent thresholds on the individual basis is difficult, if not impossible, to achieve without any prior knowledge or assumptions on the aforementioned microscopic characteristics and interactions. In addition, even properly justifying a certain shape of the emergent threshold distribution is a difficult task as it remains unclear to which extent different shapes follow from a certain composition of individual traits.

Notably, in analogy to the concept of emergent thresholds there should still exist on the micro-level a share (or number) of others that join into an action before an individual does so, too. One commonly accepted definition of such a quantity is that of a *threshold fraction*^29^ that is not assessed with respect to the entire population, but with regard to the relevant social ties of a considered individual^35,51^. The specific importance of one’s egocentric social network for decision making has recently been shown in empirical studies where individuals generally did not aim for consensus or convergence in the global population, but rather on the microscopic or group-level^19,44^. Additionally, it was observed that individuals tend to coordinate with (at least subsets of) an entire group rather a single partner^45^. This renders the use of a per-individual threshold fraction particularly useful as it determines the share of others within a group that must make a certain decision before the considered individual does so, too. In our specific case this threshold fraction is considered a fundamental trait of each individual, regardless of whether their preferences and norms favour or hinder a certain action. As such it disentangles social processes from non-social factors, such as individual preferences and norms. In contrast to the emergent thresholds, these threshold fractions may not necessarily be widespread. Rather, they might be assumed to have a narrow distribution or correspond to fixed, intuitive points, e.g. 50% (majority rule)^52^. Note that in contrast to the emergent thresholds, that measure *absolute* numbers in a global population, the threshold fraction measures the *relative* number of others in one’s egocentric social network that must make a decision before a considered individual does so, too. It thereby specifically accounts for heterogeneities in the number of each individual’s neighbors, i.e., the so-called social network’s degree distribution^53^.

Below we present a microscopic threshold model based on a previous study of cascading dynamics^29^ where individual preferences are assigned to each member of the population that then join into an action based on their threshold fractions applied to the neighborhood in their social network. We then show that such microscopic processes in fact yield an often postulated broad (but not normal-shaped) emergent threshold distribution.

## Results

### Refinement of the Model

We start by addressing the first two issues identified above, namely that for usually chosen distributions the original model predicts either no-one or the entire population to become active. As discussed above, one way to circumvent these issues is to assign certain individuals either a threshold of 0% or ≥100% such that some individuals *certainly* become active and others never become active^35^. This approach requires a constant updating of the threshold distribution and may be impracticable for many cases. Recent studies investigated the effects of either such certainly active *initiators*^42^ or never active *immune* individuals^43^ on the adoption of certain traits or behaviours via spreading dynamics on social networks. In alignment with social movement theory^40,41^ we combine these two notions and suggest to divide the population of size $N$ into three groups, namely: $A\le N$ *certainly acting* individuals^42^, *C* ≤ *N* − *A contingent* individuals and the remaining *N* − *C* − *A certainly inactive* individuals^43^. The certainly acting and contingent individuals form the group of $P=A+C$ *potentially acting* individuals. In a social movement and resource mobilization context, our three groups can for example be seen as representing adherents, potential supporters and those in opposition^40,41^.

If we have no reason to assume that the threshold distribution is different in the three groups, the original recursive formula Eq. (1) is then replaced by

2 $$R(t+1)=A+C\cdot F(R(t)).$$

The equilibria of the thus refined model are again obtained by computing the intersection of the r.h.s. of Eq. (2) with the diagonal through $(0,0)$ and $(N,N)$, Fig. 1b. It is apparent that if $A > 0$ and $P < N$ (note again that $P=A+C$), we get nontrivial equilibrium numbers of acting individuals ${R}^{\ast }\in [A,P]$. Conveniently, as *A* or *P* (and *C*) change, the new equilibria can be found without re-estimating the threshold distribution.

In order to also avoid having to redraw *F* in Fig. 1b whenever there is a variation in *A* or *C*, it is beneficial to rescale the ordinate to the unit interval, Fig. 1c. This allows us to find the equilibria for all possible combinations of *A* and *P* in the same diagram, by drawing *F* only once and just adjusting the diagonal to meet the points $(A,0)$ and $(P,1)$.

Our adjusted approach makes the application of the threshold model as an actual modeling framework more practical as it (i) produces nontrivial fixed points R*, (ii) requires the threshold distribution to be only estimated once for the entire population or a representative sample thereof, and (iii) relies on only two intuitive parameters, the size of the certainly (*A*) and potentially acting population (*P*). Recall that *A* directly relates to an immediate action or behaviour, while *P* denotes the general acceptance of or attitude towards that action.

### Estimation of the emergent threshold distribution

Having refined the threshold model to properly allow for the computation of non-trivial fixed points, we shift our focus to the second issue that relates to the threshold distribution itself. It has been established above that the emergent thresholds follow from microscopic characteristics of each individual as well as its embedding in a social context. Specifically for the latter it will turn out that the share of others, i.e., the threshold fraction, that must join into an action before a contingent individual does so, too need not be widely distributed or even heterogeneous at all across the population in order to produce a widespread distribution for the emergent threshold.

We now study how such characteristics and interactions on the micro-level determine one’s emergent threshold by using a simulation model of social contagion that has been studied in the past to model binary decisions with externalities and resulting cascading dynamics^29^. We represent each individual in the population by a node in a complex network and draw links between nodes to indicate their embedding in a social group of others (see Methods section below for details). This relates directly to the idea of a *sociomatrix* that accounts for the stronger influence that individuals to which one forms a social bond have on one’s behaviour^35^. In addition to the original formulation of this network cascade model^29^ and in agreement with the consideration put forward above we assume that *P* randomly distributed nodes form the potentially active population. Being potentially active subsumes all norms, preferences and attitudes that cause an individual to show acceptance for a considered type of behaviour. Among the *P* potentially active nodes we assume that $A\le P$ randomly distributed nodes are certainly active. In each time step each of the remaining $C=P-A$ contingent nodes *i* becomes active if more than a share $\rho \in [0,1]$ of its immediate neighbors is already active. We hence denote $\rho$ the *threshold fraction* of an individual. The resulting number or active nodes at time *t* is again denoted as *R*(*t*). Setting a common value of $\rho$ represents the most narrow distribution of actual threshold fractions that determine whether one joins into an action given that one generally supports that action at all.

**Figure 2:**
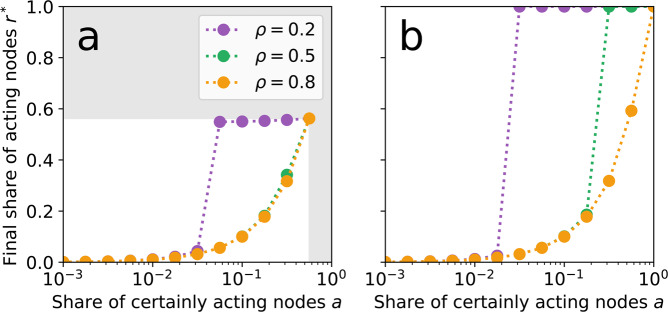
The final share of acting nodes r* in the microscopic network simulation for given shares of certainly acting nodes *a*. (**a**) With only around half the population being potentially active (i.e, $p=P/N\approx 0.56$) only a low threshold fraction ($\rho =0.2$, purple) causes large shares of the contingent nodes to act. Grey areas indicate values of r* and *a* that would exceed *p*. (**b**) If every node in the network is potentially active ($p=1$), also an intermediate threshold fraction ($\rho =0.5$, green) suffices to cause the entire population to act. In comparison with (**a**) one also observes that the transition observed for $\rho =0.2$ occurs already for smaller choices of *a*. For a large threshold fraction ($\rho =0.8$, yellow) no abrupt transition appears such that ${r}^{\ast }\,\lessapprox \,a$ for all considered choices of *a* and *p*.

We simulate cascades of nodes becoming active for two different shares of potentially active nodes $p=P/N=0.56$ (Fig. 2a) and $p=1$ (Fig. 2b), as well as for different threshold fractions $\rho \in \{0.2,0.5,0.8\}$. Figure 2 shows the final share of acting nodes ${r}^{\ast }={R}^{\ast }/N$ after the cascade stops for increasing shares of certainly acting nodes $a=A/N\le p$. For $p=0.56$ (i.e., a low share of potentially acting nodes) only small threshold fractions ($\rho =0.2$) allow for a large-scale cascade such that ${r}^{\ast }\to p$ for values of $a\,\gtrapprox \,0.05$ (Fig. 2a). In contrast, for values of $a\,\lessapprox \,0.05$ no cascade is observed and, hence, ${r}^{\ast }\,\lessapprox \,a$. Larger threshold fractions (i.e., $\rho =0.5$ or $\rho =0.8$) hinder the emergence of a cascade such that ${r}^{\ast }\,\lessapprox \,a$ for all choices of *a* (Fig. 2a). For $p=1$, cascades are also observed at a larger threshold fraction of $\rho =0.5$ but are still suppressed for $\rho =0.8$ (Fig. 2b). Furthermore, the required share of certainly acting nodes $a$ at which the system *tips* from a state with no cascades to a state with a global cascade decreases slightly with increasing $p$ (compare Fig. 2a,b). Note that specifically the role of the remaining *N* − *P* certainly inactive nodes has been studied under the term ‘*immune* nodes’ in an earlier study of spreading dynamics on networks^43^. However, in contrast to our results presented above the underlying model in this previous work^43^ assumed the share of certainly active nodes *a* to increase over time at a constant rate, thus yielding convergence to a globally stable fixed point ${r}^{\ast }=p$ for all initial choices of *a*. Hence, the major purpose of the *immune* nodes in this earlier work was to moderate the rate of convergence to that global fixed point.

**Figure 3:**
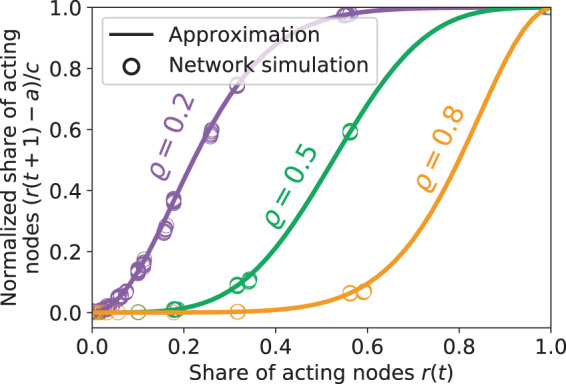
Emergent threshold distribution measured from the microscopic network simulations and the analytical approximation. For the network simulations only those points where the system is close to equilibrium, i.e. $t\in \{0,{t}_{max}-1\}$, are shown. For all shown choices of threshold fractions $\rho$, the approximation matches well with the network simulations.

To estimate an emergent threshold distribution as required for the Granovetter-type threshold model we now evaluate $r(t)=R(t)/N$ against $(r(t+1)-a)/c$ (with $c=C/N=(P-A)/N$) from the network simulations. Figure 3 shows the results if the network cascade is close to equilibrium, i.e., for $t=0$ or $t={t}_{max}-1$, where *t*_*max*_ is the time at which the cascade stops. We observe the formerly postulated broad distribution of emergent thresholds as a result of the microscopic interactions at narrowly distributed threshold fractions $\rho \in \{0.2,0.5,0.8\}$ given a generally positive (*P* nodes) or negative (*N* − *P* nodes) attitude towards the considered behavior. This implies that individuals with a high emergent threshold may not necessarily be more reluctant to join into an action, it could simply mean that they are located at a more peripheral position in the network.

By approximating the number of active, *a*_*i*_, and inactive neighbors, *b*_*i*_, of a node *i* as coming from a common multinomial distribution that only depends on the number of neighbors ${k}_{i}={a}_{i}+{b}_{i}$ and the overall share of active nodes $r(t)$, we derive an analytical approximation of the emergent threshold distribution *F* (note that for brevity we omit the dependence of $r(t)$ on *t*) as

3 $$F(r)=1-\exp (\,-\,K)\,\mathop{\sum }\limits_{{b}_{i}=0}^{\infty }\,\frac{{(K-Kr)}^{{b}_{i}}}{{b}_{i}!}\,\mathop{\sum }\limits_{{a}_{i}=0}^{[\frac{{\rho b}_{i}}{1-\rho }]}\,\frac{{(Kr)}^{{a}_{i}}}{{a}_{i}!}.$$

here, $K={\sum }_{i}\,{k}_{i}/N$ denotes the average degree (i.e., number of neighbors) of nodes in the network (see Methods section below and the Supplementary Information for a full derivation of Eq. (3)). Note that the second factor in Eq. (3) can be further approximated by an incomplete gamma function. We find that (close to equilibrium) Eq. (3) aligns very well with the network simulations for small ($\rho =0.2$), medium ($\rho =0.5$) and large ($\rho =0.8$) fractional thresholds (Fig. 3) and thus complements previously proposed approximations that primarily held for small to medium values^42^. For the transient phase the approximation still estimates the emergent thresholds well for small and large choices of $\rho$ but decreases in quality for intermediate values (see Supplementary Information). This is mainly caused by the clustering of active and inactive nodes. An extension of the above approximation that accounts for such factors, e.g., via pair approximations^54,55^ or moment generating functions^29^, is beyond the scope of this work and remains as a subject for future research. In summary, Eq. (3) gives a good estimation of an emergent macroscopic distribution that fulfills the initially postulated broad shape^35^ while emerging from a subsumed set of preferences as well as a single common threshold fraction $\rho$. In addition, using a single distribution *F* has the advantage of being independent of the share of certainly and potentially acting nodes. As such it only needs to be estimated once while changing preferences (i.e., varying *A* and *P*) are incorporated into shifting the diagonal line that is used to estimate the fixed points (see again Fig. 1c).

### Comprehensive analysis and social tipping

**Figure 4:**
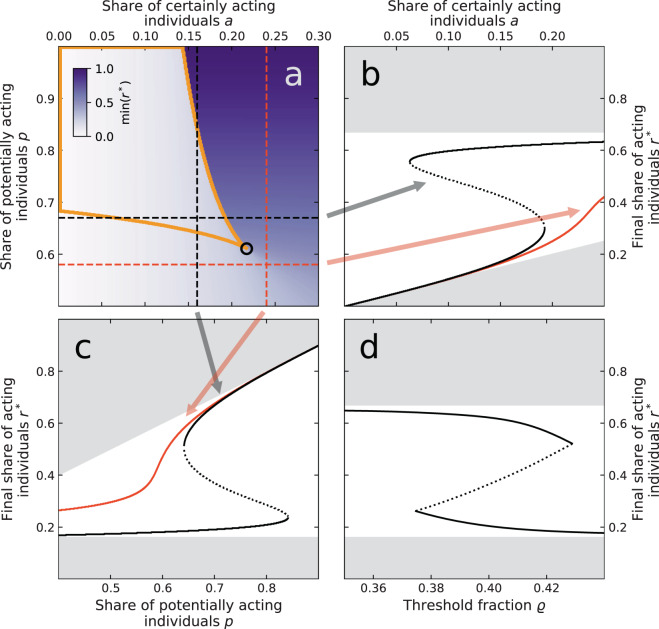
Bifurcation analysis and hysteresis of the refined Granovetter model with an emergent threshold distribution as given by the analytical approximation. (**a**) Smallest stable fixed point min(r*) for different shares of certainly acting $a$ and potentially acting individuals $p$. The black circle denotes a cusp-bifurcation. Black dashed horizontal/vertical lines correspond to the diagrams in (**b**,**c**) that show a saddle-node bifurcation. For (**b**–**d**), solid (dotted) lines indicate stable (unstable) fixed points r*. Grey shading indicates those areas where ${r}^{\ast }\notin [a,p]$ and that can thus not be reached. The yellow circled area in (**a**) indicates the bistable regime. Red dashed horizontal/vertical lines in (**a**) correspond to values of *p* and *a* at which no bifurcation is observed and thus r* varies smoothly in (**b**,**c**). (**d**) Shows the bifurcation diagram in the threshold fraction $\rho$. Fixed parameters are: $a=0.16$ for (**c**) ($a=0.24$ for the red curve) and (**d**), $p=0.67$ for (**b**) ($p=0.58$ for the red curve) and (**d**), and $\rho =0.4$ for (**a**–**c**).

From the approximate emergent threshold distribution *F* in Eq. (3) we estimate the fixed points r* of the refined threshold model for different choices of *a*, *p* (or $c=p-a$), and $\rho$ by solving $(r-a)/c=F(r)$ (i.e., intersecting the diagonal line with *F*). We either identify two stable and one unstable fixed points, or one globally stable fixed point r*. Figure 4a shows the value of the smallest stable fixed point ${\rm{\min }}({r}^{\ast })$. We find a sharp increase in its value for certain values of $0.15\,\lessapprox \,a\,\lessapprox \,0.22$ and $p\,\gtrapprox \,0.5$ hinting at a saddle-node bifurcation. Figure 4b,c show that saddle-node bifurcation at varying values of *a* and *p*, respectively. As the saddle-node bifurcation, and correspondingly also hysteresis, emerges in both parameters, the model consequently displays a cusp bifurcation as well (see black circle in Fig. 4a). For fixed values of *a* or *p* below the cusp-point the final share of acting individuals r* thus varies only smoothly with the respective other free parameter (red lines in Fig. 4b,c). In contrast, fixing either *a* or *p* to values above the cusp-point can cause the system to rapidly shift from a stable state with low r* to a stable state with high r* (and vice versa) as the corresponding bifurcation point in the remaining free parameter is crossed (black lines in Fig. 4b,c). Notably, the model shows hysteresis also within a band of possible threshold fractions, Fig. 4d.

In summary, our model conceptually shows what has formerly been termed *social tipping*, i.e., a process where, for a given population, a small change in the size of a dedicated minority can have a large effect^19,21,56^. In our specific case, for a given value of *a* or *p* a small change in the respective other parameter suffices to largely increase (or decrease) the share of finally acting individuals r*. Complementing recent theoretical and numerical studies of spreading processes on networks that either varied the size of the initiating minority^42^ or the so-called immune group of inactive nodes^43^ our model shows a bistable regime that is necessary for the emergence of hysteresis. This implies that once the system has tipped it sustains its state of high (low) shares of acting individuals r* even if *a* or *p* were to be reduced (increased) again. By incorporating both, initiating and immune groups, our model additionally gives rise to a previously undetected cusp bifurcation as well.

Remarkably, the critical size of the dedicated minority at which the system undergoes a fold bifurcation (Fig. 4a,b) has recently been empirically estimated to lie in the range $0.21\,\lessapprox \,a\,\lessapprox \,0.25$ which is consistent with the results of our model^19^. Moreover, critical minority group sizes of around 20 percent have also been discussed with respect to the Pareto principle^57^ which has recently been reframed as *the law of the vital few* to discuss matters of sustainability transformations and social tipping^58^.

## Discussion

We have proposed a refined version of the original Granovetter threshold model^35^ that addresses a set of issues that, so far, have hindered its application as a conceptual modeling tool. Specifically, we propose to divide the considered population of size *N* into three classes (certainly, potentially, and certainly not acting individuals) of different sizes $A\le P$, $P\le N$, and $N-P$. In addition, we propose a threshold distribution that emerges from microscopic interactions between individuals on a social network. This distribution solely depends on the average connectivity *K* of individuals and a common threshold fraction $\rho$ to join into an action given that their individual preferences and attitudes are already favourable with respect to that action. The four parameters of our refined model are of intuitive nature and allow for a systematic evaluation of its dynamics in terms of a bifurcation analysis (except for $K$ which only needs to be chosen sufficiently larger than zero, i.e., $K\gg 0$, see Supplementary Information for details). As in the original threshold model, an estimation of the fixed points can be obtained by (graphically) intersecting the diagonal line defined by *a* and *p* with the emergent threshold distribution *F*. The three crucial parameters *a*, *p*, and $\rho$ all cause a saddle-node bifurcation which is a prototypical mechanism behind tipping points in many other systems, such as in ecology^59,60^ or the climate system^61,62^, as well. It thus makes the model a promising tool to study the emerging field of social tipping^19,21,56^ where *little things can make a big difference*^63^ and minority groups can trigger large shares of a population to engage in collective action.

Our revised model describes multiple forms of collective behaviors, including social movements and crowd-like behaviors. For both such behaviors, norms are directly called upon to structure individual likelihood to engage in actions while also observing the actions of others around them. Importantly, there are differences in the speed of the process. For crowds the observation of social members is made relatively quickly, as are the decisions to participate in the actions. In contrast, these processes can be much slower for social movements. For both cases, however, we identify three time scales that are underlying our refined threshold model. We assume that the microscopic threshold fractions change at the slowest time scale (usually years to decades), as these are attributed to the unique identity of an individual (which may be less prone to sudden external shocks). In contrast, the classification into *certainly* or *contingently* active individuals varies on intermediate time scales (months to years) as changes in the environment (such as financial shocks or the exposition to increasing extreme weather events) are beyond an individual’s own agency and can trigger sudden changes in attitudes^64^. The social dynamics modelled here, i.e., the observation of others and the joining into an action, are happening on the fastest time scale (days to months) as frequent social interactions are common among members of any given society.

Most parameters of the refined model may be readily measurable in a variety of applications. Attitudes that determine *p* could be estimated from surveys or existing panel data. The share of certainly acting individuals *a* could be given by those in the population that inevitably need to act, e.g., migrate as a consequence of climate change impacts^65,66^. For the average degree *K* it may often suffice to set it to a reasonable number, e.g., Dunbar’s number that suggests a cognitive limit to the number of people with whom an individual can maintain a persistent social relationship^67^ (see Supplementary Information for details). The threshold fraction $\rho$ could then either remain as a free parameter of the model or be set to fixed intuitive points such as 50% (majority rule) or 20% (Pareto principle^57,58^). Furthermore, the model also allows for changes in its parameters over time, such that r* can be estimated as a time-dependent variable, possibly causing the system to tip back and forth between its two possible stable states. In that sense the respective parameters can be incorporated into the system’s internal dynamics as slowly changing variables.

Future work should concentrate on collecting data for the different parameters and then consequently test and calibrate the model against historical test cases. One specific challenge that lies within such an endeavor is the estimation of appropriate (relative) time scales at which the parameters and the internal variables change. In addition, appropriate early-warning indicators^62,68,69^ should be applied to study the existence of precursory signals for the transgression of a social tipping point, i.e., bifurcation, in our model. Some of these indicators would require a further extension of the model such that individuals may also spontaneously become active with a low probability even if their threshold fraction is not transgressed (or vice versa). We further acknowledge that up to now a proposal for an emergent threshold distribution has only been derived analytically for the case of an Erdős—Rényi random network^70^. While this lays good groundwork, the threshold distribution should also be explored for topologies (such as *scale*-*free*^71^ and *small*-*world* networks^72^) that more closely mimic those of real-world social systems. Hence, even though our proposed approximation of the emergent threshold distribution holds well if the system is well-mixed and close to a fixed point, more elaborate methods, e.g., pair approximations^55^ and moment generating function approaches^29^, should be used to predict the model’s dynamics for more general network topologies and during transient phases as well. Ultimately, the model should be applied as a conceptual modeling tool, e.g., to make qualitative statements on the possibility for social tipping with respect to issues of global change or sustainability transformations^12,73,74^ under different scenarios.

## Methods

### Network cascade model

For the microscopic network simulation we consider an Erdős—Rényi random network^70^ with $N=100\,000$ nodes and a linking probability of $\ell =9\cdot {10}^{-5}$ resulting in an average degree of $K=10$. We vary the number of certainly acting nodes *A* logarithmically between 1 and *N* and the number of potentially acting nodes logarithmically between $A$ and $N$. For each setting of $A$ and $P$ (and fixed values of the threshold fraction $\rho$ as given in Fig. 2) we create an ensemble of $n=100$ networks and randomly assign $P$ out of the $N$ nodes as potentially active. Out of those $P$ nodes we then randomly assign $A$ certainly acting nodes. The model then runs in discrete time steps $t$. In each time step, every potentially active, yet inactive, node *i* becomes active if its share of active neighbors exceeds the threshold fraction $\rho$. All nodes update their status synchronously at each time step. The simulation stops if the number of newly activated nodes at time *t* equals zero, i.e., if $R(t-1)=R(t)$. Note that our model is based on previous works that implemented a simpler version of a cascade model that did not account for a distinction in potentially active and certainly inactive nodes^29^.

### Approximation of the emergent threshold distribution

The approximate emergent threshold distribution *F* in Eq. (3) is derived by assuming that for each individual *i* the number of active *a*_*i*_ and inactive neighbors *b*_*i*_ are distributed according to a common multinomial distribution, giving

4 $$F(R)=\sum _{\begin{array}{c}{a}_{i} > \rho ({a}_{i}+{b}_{i})\\ {a}_{i}\le R\\ {b}_{i}\ge 0\\ {b}_{i}\le P{\prime} \end{array}}\,(\begin{array}{l}R\\ {a}_{i}\end{array})(\begin{array}{l}P{\prime} \\ {b}_{i}\end{array}){\ell }^{{a}_{i}}{\ell }^{{b}_{i}}{(1-\ell )}^{R-{a}_{i}}{(1-\ell )}^{P{\prime} -{b}_{i}}.$$

*P*′ = *N* − 1 − *R* denotes the number of inactive individuals that are not the considered *i*, as one’s own level of activity is not accounted for. $\ell$ is the linking probability of the Erdős—Rényi network. Equation (3) follows from Eq. (4) by setting $R=\lfloor rN\rfloor$, substituting the binomial distributions by two Poisson distributions with expectation values ${\lambda }_{a}=Kr$ and ${\lambda }_{b}=K-Kr$ and assuming that $N\gg K$. A step-by-step derivation of Eq. (3) is given in the Supplementary Information.

## Supplementary information

Supplementary Information.
