# Supplementary material for: A network-based microfoundation of Granovetter’s threshold model for social tipping
Source: Sci Rep. 2020 Jul 8;10:11202. doi: 10.1038/s41598-020-67102-6 (PMC7343878; doi:10.1038/s41598-020-67102-6)
Supplement: Supplementary file 1 — Supplementary Information. [file 41598_2020_67102_MOESM1_ESM.pdf]

A network-based microfoundation of Granovetter's threshold model for social tipping — Supplementary Information

Marc Wiedermann[1,*], E. Keith Smith[2,3], Jobst Heitzig[1], and Jonathan F. Donges[4,5]

[1]FutureLab on Game Theory & Networks of Interacting Agents, Complexity Science, Potsdam Institute for Climate Impact Research, Member of the Leibniz Association, P.O. Box 60 12 03, 14412 Potsdam, Germany
[2]GESIS — Leibniz Institute for the Social Sciences, Member of the Leibniz Association, Unter Sachsenhausen 6-8, 50667 Cologne, Germany
[3]Institute of Science, Technology and Policy, ETH Zurich, Zurich, Switzerland
[4]FutureLab Earth Resilience in the Anthropocene, Earth System Analysis, Potsdam Institute for Climate Impact Research, Member of the Leibniz Association, P.O. Box 60 12 03, 14412 Potsdam, Germany
[5]Stockholm Resilience Centre, Stockholm University, Kräftriket 2B, 114 19 Stockholm, Sweden
[*]marcwie@pik-potsdam.de

Derivation of the macroscopic approximation for the emergent threshold distribution

As stated in the main manuscript, we divide the N individuals in a given population into three groups of certainly active (with size A), contingently active (with size C) and certainly inactive (with size $N-A-C$) individuals. In order to explain and derive the emergent broad threshold distribution F we utilize a microscopic network model of cascading dynamics in which each node represents one of the N individuals in the population. The network is constructed as an Erdős-Rényi network with a fixed number of nodes N and a linking probability ℓ[1]. Nodes are randomly assigned once and for all to either of the three groups outlined above. A contingent node i is active at the next time point $t+1$ iff the number of its active neighbors a_i at the current time point t exceeds a certain share ρ (denoted as i's individual threshold fraction) of its total number of neighbors k_i, i.e., if

$$a_i > \rho k_i = \rho \cdot (a_i + b_i). \tag{1}$$

Here, b_i denotes the number of currently inactive neighbors of node i.

Now, for a given number of acting nodes $R(t)$, we aim to approximate the number of acting nodes $R(t+1)$ at the next time step (analogously to Eq. (2) of the main manuscript) by

$$R(t+1) = A + CF(R(t)) \tag{2}$$

for some function F to be determined. If we interpret $F(R(t))$ as the probability for each contingent node i to be active at time $t+1$ for a given number of acting nodes $R(t)$ and make the approximation that the distribution of active and inactive nodes is the same in the neighbourhoods of all nodes, then this probability follows from a multinomial distribution as

$$F(R(t)) = \sum_{\substack{a_i \geq 0 \\ a_i > \rho\cdot(a_i+b_i) \\ a_i \leq R(t) \\ a_i+b_i \leq N-1 \\ b_i \geq 0 \\ b_i \leq N-R(t)-1}} \binom{R(t)}{a_i} \binom{N-R(t)-1}{b_i} \ell^{a_i} \ell^{b_i} (1-\ell)^{R(t)-a_i} (1-\ell)^{N-R(t)-1-b_i}. \tag{3}$$

The first condition $a_i \geq 0$ under the sum forbids negative numbers of active nodes and the second condition follows from Eq. (1). The latter imposes a stronger constraint on a_i such that we obtain a lower bound of a_i as

$$
\begin{aligned}
& a_i \geq 0 \wedge a_i > \rho \cdot (a_i + b_i) && (4)\\
\Leftrightarrow\ & a_i > \frac{b_i \rho}{1-\rho} && (5)\\
\Leftrightarrow\ & a_i \geq \left\lfloor \frac{\rho b_i}{1-\rho} \right\rfloor + 1. && (6)
\end{aligned}
$$

For setting an upper bound on a_i we utilize the third and fourth condition in the sum of Eq. (3). The third condition indicates that the number of active neighbors a_i of i can not exceed the total number of active nodes or individuals $R(t)$. The fourth condition forbids the degree $k_i = a_i + b_i$ of node i to exceed its maximum possible value $N-1$ (as we no not allow for self-loops). Both conditions together give an upper bound of a_i as

$$
\begin{aligned}
& a_i \leq R(t) \wedge a_i + b_i \leq N-1 && (7)\\
\Rightarrow\ & a_i \leq R(t) \wedge a_i \leq N-1-b_i. && (8)
\end{aligned}
$$

However, the last condition in the sum of Eq. (3) states that $N-1-b_i \geq R(t)$ and thus the upper bound of a_i reduces to

$$a_i \leq R(t). \tag{9}$$

Ultimately, the upper and lower bounds of b_i are given directly by the last two conditions in the sum of Eq. (3). Plugging all upper and lower bounds back into Eq. (3) yields

$$F(R(t)) = \sum_{b_i=0}^{N-R(t)-1} \binom{N-R(t)-1}{b_i} \ell^{b_i} (1-\ell)^{N-R(t)-1-b_i} \sum_{a_i=\left\lfloor \frac{\rho b_i}{1-\rho} \right\rfloor+1}^{R(t)} \binom{R(t)}{a_i} \ell^{a_i} (1-\ell)^{R(t)-a_i} \tag{10}$$

From here, we utilize a set of approximations to further simplify the above expression. First, we introduce $r(t) \in [0,1]$ as the fraction of active nodes at time t, such that the number of active nodes reads $R(t) = \lfloor r(t)N \rfloor$. Plugging this back into Eq. (10) yields

$$F(r(t)) = \sum_{b_i=0}^{N-\lfloor r(t)N \rfloor-1} \binom{N-\lfloor r(t)N \rfloor-1}{b_i} \ell^{b_i} (1-\ell)^{N-\lfloor r(t)N \rfloor-1-b_i} \sum_{a_i=\left\lfloor \frac{\rho b_i}{1-\rho} \right\rfloor+1}^{\lfloor r(t)N \rfloor} \binom{\lfloor r(t)N \rfloor}{a_i} \ell^{a_i} (1-\ell)^{\lfloor r(t)N \rfloor-a_i}. \tag{11}$$

Furthermore, since we generally consider large N and small ℓ, we approximate the two binomial distributions by the respective Poisson distributions with shape parameters λ_a and λ_b given as

$$
\begin{aligned}
\lambda_a &= \ell \lfloor N r(t) \rfloor && (12)\\
\lambda_b &= \ell (N - \lfloor N r(t) \rfloor - 1) && (13)
\end{aligned}
$$

Making use of the fact that the linking probability in an Erdős-Rényi graph can also be expressed in terms of the network's average degree K as $\ell = K/(N-1)$ and additionally using that $N \gg K$ such that $\ell \approx K/N$ we can approximate both shape parameters as

$$
\begin{aligned}
\lambda_a &= \frac{K \lfloor N r(t) \rfloor}{N-1} \approx K r(t) && (14)\\
\lambda_b &= \frac{K(N - \lfloor N r(t) \rfloor - 1)}{N-1} \approx K(1-r(t)). && (15)
\end{aligned}
$$

Hence, we approximate Eq. (11) (under omittance of the explicit time dependence of $r(t)$) to yield

$$F(r) \approx \sum_{b_i=0}^{N-\lfloor rN \rfloor -1} \frac{(K-Kr)^{b_i}}{b_i!} \exp(-K+Kr) \sum_{a_i=\left\lfloor \frac{\rho b_i}{1-\rho} \right\rfloor +1}^{\lfloor rN \rfloor} \frac{(Kr)^{a_i}}{a_i!} \exp(-Kr) \tag{16}$$

$$= \sum_{b_i=0}^{N-\lfloor rN \rfloor -1} \frac{(K-Kr)^{b_i}}{b_i!} \exp(-K+Kr) \left(\sum_{a_i=0}^{\lfloor rN \rfloor} \frac{(Kr)^{a_i}}{a_i!} \exp(-Kr) - \sum_{a_i=0}^{\left\lfloor \frac{\rho b_i}{1-\rho} \right\rfloor} \frac{(Kr)^{a_i}}{a_i!} \exp(-Kr) \right) \tag{17}$$

$$= \exp(-K) \left(\underbrace{\sum_{b_i=0}^{N-\lfloor rN \rfloor -1} \frac{(K-Kr)^{b_i}}{b_i!}}_{\exp(K-Kr) \text{ for } N \gg K} \underbrace{\sum_{a_i=0}^{\lfloor rN \rfloor} \frac{(Kr)^{a_i}}{a_i!}}_{\exp(Kr) \text{ for } N \gg K} - \sum_{b_i=0}^{N-\lfloor rN \rfloor -1} \frac{(K-Kr)^{b_i}}{b_i!} \sum_{a_i=0}^{\left\lfloor \frac{\rho b_i}{1-\rho} \right\rfloor} \frac{(Kr)^{a_i}}{a_i!} \right) \tag{18}$$

$$\approx 1 - \exp(-K) \sum_{b_i=0}^{N-\lfloor rN \rfloor -1} \frac{(K-Kr)^{b_i}}{b_i!} \sum_{a_i=0}^{\left\lfloor \frac{\rho b_i}{1-\rho} \right\rfloor} \frac{(Kr)^{a_i}}{a_i!}. \tag{19}$$

We will now eliminate the explicit dependency on N in the first sum by setting

$$F(r) \approx 1 - \exp(-K) \left(\sum_{b_i=0}^{\infty} \frac{(K-Kr)^{b_i}}{b_i!} - \sum_{b_i=N-\lfloor rN \rfloor}^{\infty} \frac{(K-Kr)^{b_i}}{b_i!} \right) \sum_{a_i=0}^{\left\lfloor \frac{\rho b_i}{1-\rho} \right\rfloor} \frac{(Kr)^{a_i}}{a_i!}. \tag{20}$$

We can now estimate an upper bound of the second sum in the brackets as

$$\exp(-K) \sum_{b_i=N-\lfloor rN \rfloor}^{\infty} \frac{(K-Kr)^{b_i}}{b_i!} \underbrace{\sum_{a_i=0}^{\left\lfloor \frac{\rho b_i}{1-\rho} \right\rfloor} \frac{(Kr)^{a_i}}{a_i!}}_{\leq \exp(Kr)} \leq \exp(-K+Kr) \sum_{b_i=N-\lfloor rN \rfloor}^{\infty} \frac{(K-Kr)^{b_i}}{b_i!} \tag{21}$$

$$\leq \frac{(K-Kr)^{N-\lfloor rN \rfloor}}{(N-\lfloor rN \rfloor)!} \approx \left(\frac{\exp(1)(K-Kr)}{(N-\lfloor rN \rfloor)!} \right)^{N-\lfloor rN \rfloor} \to 0 \text{ for } r<1 \text{ and } K \ll N. \tag{22}$$

For $r=1$ the two sums over b_i in Eq. (20) have the same limits and thus the difference between the two vanishes. Similarly, the sum over b_i in Eq. (19) vanishes such that Eq. (19) equals Eq. (20) for $r=1$ as well. Thus we can we eliminate the explicit dependence on N in the first sum of Eq. (19) for all valid choices of r and ultimately obtain

$$F(r) \approx 1 - \exp(-K) \sum_{b_i=0}^{\infty} \frac{(K-Kr)^{b_i}}{b_i!} \sum_{a_i=0}^{\left\lfloor \frac{\rho b_i}{1-\rho} \right\rfloor} \frac{(Kr)^{a_i}}{a_i!}. \tag{23}$$

For the sake of completeness and to demonstrate the validity of our approximation, we consider the two limiting cases $r \to 0$ and $r \to 1$. For the first case, i.e., $r \to 0$, it follows that $(Kr)^{a_i} \to 1$ if $a_i = 0$ and $(Kr)^{a_i} \to 0$ if $a_i > 0$, hence

$$\lim_{r \to 0} \sum_{a_i=0}^{\left\lfloor \frac{\rho b_i}{1-\rho} \right\rfloor} \frac{(Kr)^{a_i}}{a_i!} = 1 \tag{24}$$

$$\Rightarrow \lim_{r \to 0} F(r) = 1 - \lim_{r \to 0} \exp(-K) \sum_{b_i=0}^{\infty} \frac{(K-Kr)^{b_i}}{b_i!} = 1 - \lim_{r \to 0} \exp(-Kr) = 0. \tag{25}$$

For $r \to 1$ we obtain that

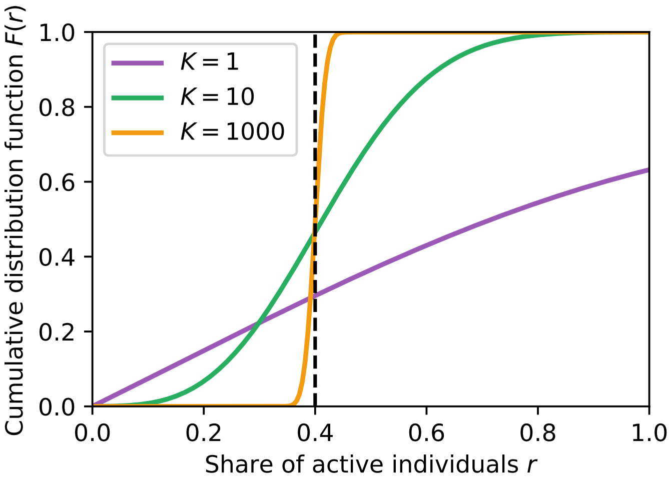

Figure 1. Analytical approximation of the cumulative distribution function F of emergent thresholds as given in Eq. (23) for $\rho = 0.4$ (dashed line) and increasing K. For large values of K, F approaches a step-function such that $F(r) = 0 \; \forall \; r < \rho$ and $F(r) = 1 \; \forall \; r > \rho$.

$$\lim_{r \to 1} (K - Kr)^{b_i} \begin{cases} = 1 \text{ if } b_i = 0 \\ \to 0 \text{ else} \end{cases} \tag{26}$$

$$\Rightarrow \lim_{r \to 1} F(r) = 1 - \exp(-K) \sum_{a_i = 0}^{0} \frac{(K)^{a_i}}{a_i!} = 1 - \exp(-K) \to 1 \text{ if } K \gg 0. \tag{27}$$

In summary, we thus obtain that $F(r) \to 0$ for $r \to 0$ as expected and $F(r) \to 1$ for $r \to 1$ and $K \gg 0$. For the special case of small average degree $K = \mathcal{O}(0)$ the network becomes increasingly disconnected. Thus, some contingent nodes might be isolated from the rest of the network and thus never become active. As a consequence one obtains the correction $\exp(-K)$ in the limit $\lim_{r \to 1} F(r)$. Notably for $K = 0$ we correctly obtain $F(r) = 0 \; \forall \; r \in [0, 1]$ as all nodes are isolated and no contagious dynamics or cascades can occur.

Further assessment, limiting cases and influence of the average degree on the existence of social tipping points

We now estimate a reasonable range for the average degree K in a sense that the model qualitatively recaptures the dynamics that are presented in the main manuscript. We also show that the choice of $K = 10$ that was used in the main manuscript lies well within that reasonable range.

Limiting cases of the average degree

We start with investigating the influence of the average degree K on the fixed points r^* that are obtained from the macroscopic approximation of our model. Recall from above that for $K = 0$, we find that $F(r) = 0$. Similarly, we argue that for $K \to \infty$ (or $K \to N$ in the case of a finite number of nodes N) the network becomes increasingly connected and approaches the complete graph. In that case, all nodes are neighbors to each other and, hence, nodes become active if the total share of active nodes r (i.e., $r(t)$) simply exceeds the individual threshold fraction ρ. Thus, we expect that $\lim_{K \to \infty} F(r) \to \Theta(\rho)$ where $\Theta(\cdot)$ is the Heaviside step-function.

We confirm this expectation numerically by computing F for increasing values of K, Fig. 1. We observe that with increasing K the approximation first approaches $F(r = 1) = 1$ (see also Eq. (27)) and in a second step approaches a step function with its critical point at the individual threshold fraction ρ. Thus, we conclude that there exists an intermediate range of K for which the emergent threshold distribution has the expected and postulated broad shape[2] such that the model produces the dynamics (in terms of cusp- and saddle-node bifurcations) as described in the main manuscript.

Influence of the average degree on the existence and location of the cusp-point

To gain further insights on what might constitute a reasonable range of the average degree K, we study the position of the cusp-point $x_c = (a_c, p_c)$ (like it is for instance displayed in Fig. 4a of the main manuscript) for varying choices of K (and fixed

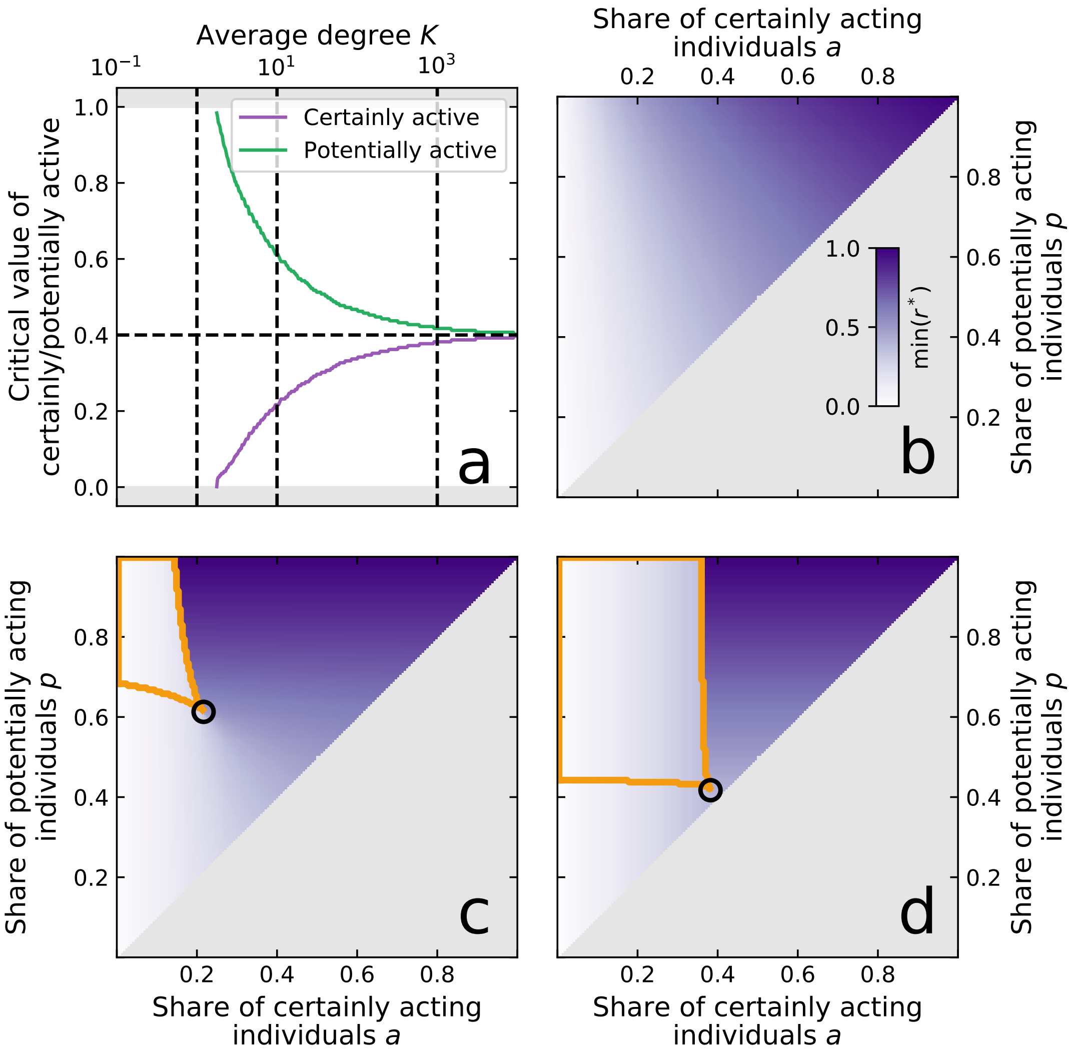

Figure 2. The location of the cusp-point $x_c = (a_c, p_c)$ depending on the choice of the average degree K for a fixed individual threshold fraction $\rho = 0.4$. (a) The critical shares a_c of certainly acting (purple) and p_c of potentially acting individuals (green) as a function of the average degree K. The horizontal dashed-line indicates the value of $\rho = 0.4$ that is approached by a_c and p_c with increasing K. The vertical lines indicate the three choices of K for which the value of the smallest stable fixed point $\min(r^*)$ is displayed in (b) ($K = 1$), (c) ($K = 10$) and (d) ($K = 1000$), respectively. The black circle in (c) and (d) indicates the cusp-point x_c. Grey areas in all panels indicate illicit combinations of a and p. The yellow circled area in (c) and (d) indicates the bistable regime.

$\rho = 0.4$). We therefore estimate numerically the critical shares of certainly active and potentially active nodes a_c and p_c at which the cusp-bifurcation occurs, Fig. 2a.

We find that for very low values of K the cusp-point vanishes, while for large K we obtain that $a_c \to \rho$ from below and $p_c \to \rho$ from above, Fig. 2a. Thus, the cusp-point moves from the upper left corner of the parameter plane ($a_c = 0$ and $p_c = 1$) to the edge of the allowed parameter regime bounded by $a \leq p$ and approaches $a_c = p_c = \rho$. Hence, for $K \to \infty$ the cusp-point lies directly on the edge of the parameter plane.

Fig. 2b,c,d show (in analogy to Fig. 4a of the main manuscript) the value of the smallest stable fixed point $\min(r^*)$ for different shares of certainly and potentially active individuals a and p as well as fixed values of $K = 1$ (Fig. 2b), $K = 10$ (Fig. 2c), and $K = 1000$ (Fig. 2d), respectively. For $K = 1$ the smallest stable fixed point (which is the only stable fixed point for all choices of parameters) varies smoothly with changing a and p and, hence, no bifurcation (and no tipping) occurs. For $K = 10$ we find intermediate values of a_c and p_c and thus the cusp-point can be crossed in both the direction of a and p. Note that Fig. 2c corresponds to Fig. 4a in the main manuscript, but shows the entire valid parameter space. For $K = 1000$ (Fig. 2d) the cusp-point moves to the outer diagonal edge of the parameter plane. Consequently the system approaches a state (that

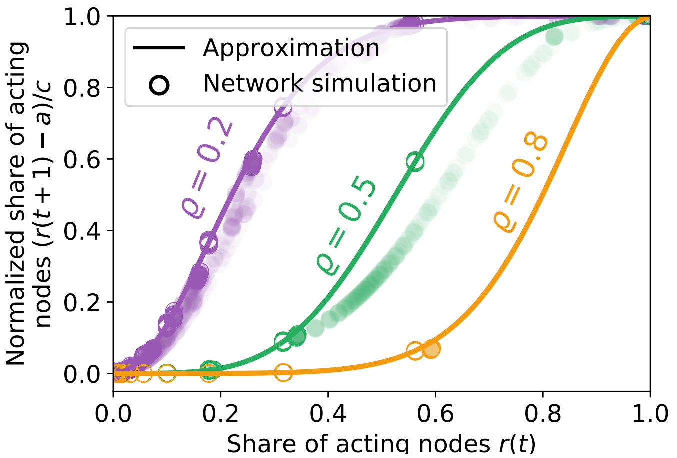

Figure 3. Same as Figure 3 of the main manuscript but including pairs of $r(t)$ and normalized $r(t+1)$ for the transient phase of the network simulation (where $t = 1, 2, \ldots t_{max} - 2$) shown as semi-transparent scatter. We note that the approximation looses some accuracy for the transient phase if one goes from individual threshold fractions $\rho = 0.2$ to $\rho = 0.5$. For $\rho = 0.8$ cascading dynamics are not very likely and only occur if the share of certainly acting nodes in the network simulation is sufficiently high. Hence, the transient phase becomes very short as either no cascade starts or all nodes almost immediately become active once the share of certainly acting nodes is large enough. Therefore, only few additional data points for the transient phase are observed for $\rho = 0.8$ and the analytical approximation remains to match well with the numerics.

would be reached if K were to be increased further) where it becomes monostable for $a < p < \rho$ or $a > \rho,\ p > \rho$ and bistable for $a < \rho,\ p > \rho$ (yellow boundaries in Fig. 2d).

Additionally, we estimate numerically a critical value of K_c as the smallest value of K for which the cusp-point x_c still exists and obtain $K_c \approx 1.801$ for $\rho = 0.4$.

Hence, for all choices of K that lie in a range that is reasonable for real-world applications the model produces qualitatively similar results in alignment with the results shown in Fig. 4 of the main manuscript. For the results put forward in the main manuscript we deliberately choose to set $K = 10$ to allow for a sufficiently long transient phase of the network simulations. Even more realistic choices of K, e.g., close to Dunbar's number[3], are expected to provide qualitatively similar results according to the assessment provided in this section.

Comparison between transient phases in the simulations and the analytical approximation

Fig. 3 of the main manuscript shows a comparison of the cumulative distribution of emergent thresholds F obtained from the analytical approximation and the corresponding normalized shares of acting individuals $(r(t+1)-a)/c$ (both as a function of the share of currently acting individuals $r(t)$) obtained from the numerical network model for all times where the system is close to equilibrium, i.e., $t \in \{0, t_{max} - 1\}$. Fig. 3 shows the same, but includes also the combinations of $r(t)$ and normalized $r(t+1)$ obtained for the transient phase of the network simulation, i.e., $t = 1, 2 \ldots, t_{max} - 2$. As already discussed in the main manuscript, we find that measures taken during the transient phase do not match as well with the approximation as the points that are close to equilibrium. This mainly stems from induced correlations in the neighborhood structure of active and inactive nodes as the activity spreads through the network. Since our approximation is based on a multinomial distribution we assume that the probability for two nodes to be connected is the constant value ℓ regardless whether we consider a pair of two active, two inactive or an active and an inactive node. However, during the course of the simulation the active and inactive nodes are naturally expected to form clusters such that the activity spreads along their front. Hence, the assumption of a perfect mixing of the two states induces inaccuracies when comparing our approximation with measures taken during the transient phase of the network simulation. Since our present work is not focused on capturing these dynamics during the transient phase, but (along the lines of the original threshold model[2]) only aims to estimate the final number of acting individuals r^*, we argue that the proposed approximation serves this purpose already very well (as can be seen from the good alignment of the network simulations close to equilibrium and the approximation as shown in Fig. 3 of the main manuscript and Fig. 3). Future work should, however, aim to improve the approximation also for the transient phase of the model's dynamics. One possible way to achieve this is by explicitly deriving dynamic equations not only for the share of acting individuals or nodes $r(t)$ but also for the different types of links between them using, e.g, different techniques of pair-approximations[4–6].

References

1. Erdős, P. & Rényi, A. On the Evolution of Random Graphs. In *Publication of the Mathematical Institute of the Hungarian Academy of Sciences*, 17–61 (1960).
2. Granovetter, M. Threshold Models of Collective Behavior. *Am. J. Sociol.* **83**, 1420–1443 (1978).
3. Dunbar, R. I. M. Coevolution of neocortical size, group size and language in humans. *Behav. Brain Sci.* **16**, 681–694 (1993).
4. Wiedermann, M., Donges, J. F., Heitzig, J., Lucht, W. & Kurths, J. Macroscopic description of complex adaptive networks coevolving with dynamic node states. *Phys. Rev. E* **91**, 052801 (2015).
5. Gross, T., D'Lima, C. J. D. & Blasius, B. Epidemic dynamics on an adaptive network. *Phys. Rev. Lett.* **96**, 208701 (2006).
6. Gleeson, J. P. Binary-State Dynamics on Complex Networks: Pair Approximation and Beyond. *Phys. Rev. X* **3**, 021004 (2013).
